# Effectiveness of a modified doctor–patient communication training Programme designed for surgical residents in China: a prospective, large-volume study at a single Centre

**DOI:** 10.1186/s12909-019-1776-7

**Published:** 2019-09-05

**Authors:** Song Bai, Bin Wu, Zichuan Yao, Xianqing Zhu, Yunzhong Jiang, Qing Chang, Xue Bai, Tong Tong

**Affiliations:** 1 Department of Urology, Shengjing Hospital of China Medical University, Shenyang, 110004 China; 2 Department of Graduate Medical Training, Shengjing Hospital of China Medical University, Shenyang, 110004 China; 3 Department of Student Affairs Department, Shengjing Hospital of China Medical University, 36 Sanhao Street, Shenyang, 110004 Liaoning China

**Keywords:** Doctor-patient communication, Doctor-patient relationship, Resident, Standardized patient, SEGUE

## Abstract

**Background:**

Effective doctor-patient communication (DPC) is important to improve the quality of care and treatment outcomes. This study aimed to evaluate the effectiveness of a modified DPC training programme designed for surgical residents in China.

**Methods:**

A total of 210 surgical residents from 7 specialties were recruited to participate in a communication skills training programme. The extended SEGUE scale was administered to supervisors, and a visual analogue scale (VAS) was administered to standardized patients (SPs) and trainees. Evaluations were conducted by comparing the pre-post scale scores (before, immediately after and 1 month after the programme). Training effects were assessed using the Friedman test and the intraclass correlation coefficient (ICC).

**Results:**

Compared to the extended SEGUE scale total scores before the programme, the scores both immediately after and 1 month after the program increased significantly (15.88 ± 1.93 vs. 26.40 ± 1.47 and 26.15 ± 1.36, respectively, *p* < 0.001). The scores of five of the six dimensions in the extended SEGUE scale significantly increased (p < 0.001), except for the patient’s perspective dimension score, which had no change (*p* = 0.162). With respect to this dimension, a subgroup analysis by gender indicated an increase among females (*p* < 0.001) and a decrease among males (p < 0.001). The VAS scores, which were evaluated for the SPs and trainees, increased significantly compared to the scores before the program, both immediately after and 1 month after the programme (4.31 ± 1.35 vs. 7.36 ± 1.62 and 7.34 ± 1.24, *p* < 0.001; 7.31 ± 1.25 vs. 8.39 ± 1.32 and 8.30 ± 1.24, *p* < 0.001, respectively). The consistency of the VAS between the SPs and surgical residents was 0.26 (*p* < 0.001), 0.70 (p < 0.001), and 0.70 (p < 0.001) before, immediately after and 1 month after the programme, respectively.

**Conclusion:**

This training programme improved the DPC competency of surgical residents, effectively increased the levels of satisfaction of both SPs and surgical residents, and improved the consistency of evaluation between SPs and surgical residents during doctor-patient encounters.

The registration UIN is ChiCTR1800019790 from November 28, 2018.

**Electronic supplementary material:**

The online version of this article (10.1186/s12909-019-1776-7) contains supplementary material, which is available to authorized users.

## Background

Doctor-patient communication (DPC) is defined as a special form of interpersonal communication that refers to the information and emotional communication between medical care providers and patients by means of language and behaviour. The basic ethical principles of DPC include trust, respect, equity, confidentiality, and informed consent [1]. DPC is a core skill of medical care providers [2]. Effective DPC helps to ensure that the patient adheres to medical recommendations, increases patient satisfaction, and leads to better treatment outcomes [3]. However, ineffective DPC is associated with negative experiences by patients, low compliance, medical malpractice, and negative career satisfaction for doctors [4].

In recent years, the guidelines for doctor-patient relationships have led to a shift from a doctor-centred style to a patient-centred style. In addition to professional knowledge, the DPC competency of medical care providers has also become an important indicator to measure their working ability [5]. Since 2004, the second step of the United States Medical Licensing Examination (USMLE) has involved assessing the DPC skills of medical students with standardized patients. Over the past decade, the issue of DPC in China has been subject to severe conflict [1]. Studies have shown that 98.47% of hospitals have had medical disputes and violence against medical care providers, some of whom have even been murdered by patients [6]. The number of medical students is decreasing, and only one-sixth of medical license holders have been registered at hospitals [7]. The deteriorating relationship between doctors and patients has transformed a promising career path into a dangerous job [8]. According to a national survey, more than 70% of the legal cases taken by patients against medical practitioners were associated with a perceived lack of caring and inadequate communication with the patient [1] There are many barriers between doctors and patients, including the anxiety and fear experienced by patients, unrealistic patient expectations, the workload of medical practitioners, and fears of litigation [9]. However, this situation can be largely attributed to poor doctor-patient communication [8]. Thus, it is imperative that doctors develop and improve their own communication skills to promote more effective medical practice.

Doctors are not born with excellent communication skills. Traditionally, DPC skills have been acquired either through clinical practice or by observing senior doctors [5]. Unfortunately, many studies have shown that few doctors are able to develop DPC competence through such means [10], as these skills cannot be developed automatically through experience over time. Rather, they can only be taught and trained and subsequently mastered over a period of years [11]. DPC training programmes were introduced in China from the Western world over 10 years ago. For example, the SEGUE framework [12] and the Calgary-Cambridge Guide [13] have been modified and adapted for Chinese culture. Nevertheless, previous research has indicated that DPC training programmes are limited both in medical schools and post-graduate education in China and that they place greater emphasis on theoretical knowledge while lacking practical training in communication skills [14]. In particular, no training programmes have been designed specifically for surgical residents. In addition, studies describing DPC skills training programmes usually report only immediate post-training assessments. Therefore, there is an urgent need to explore a set of practical training-based DPC programmes that could be proven to be practically effective and widely applicable for surgical residents. Therefore, we constructed a modified DPC training programme derived from the SEGUE framework that may address this dilemma. The purpose of this study was to evaluate the effectiveness of the modified DPC training programme for surgical residents in China. This study included longitudinal post-training assessments both immediately after the programme and 1 month after the programme, and the results were more reliable than those of studies that have relied only on immediate post-training assessments.

## Methods

### Study design and participants

This prospective study had a pre-post, single-arm intervention design and was conducted in our hospital between December 2018 and January 2019. We recruited 210 surgical residents from 7 specialties who satisfied the inclusion criteria. The scale was utilized to evaluate the participants by comparing the pre-post scale scores (before the programme, immediately after the programme and 1 month after the completion of the programme). The training programme was completed within two weeks.

### Inclusion and exclusion criteria

The inclusion criteria were as follows: occupationally active surgical residents employed in our hospitals who were willing to participate in the training programme and assessment procedure. The following exclusion criteria were used: surgical residents who had previously participated in a psychological training programme or similar curriculum and residents who completed less than 80% of the training programme.

### Programme curriculum

The development of the doctor-patient communication training programme (DPCTP) was based on a literature search, an expert seminar, and a questionnaire survey of residents’ requirements. The objective of the programme was that after completion of the workshop, the participants would be able to apply efficient DPC skills in routine or challenging encounters with patients with or without family members.

The programme was learner-centred, skills-focused, and practice-oriented. A multi-model teaching strategy in small groups (i.e., five residents) was employed. Each discussion group was assigned a supervisor who facilitated the course in his or her capacity as a mentor, teacher, or course organizer. The supervisors were either professional attending surgeons with substantial experience in DPC or had previously undergone training in a similar DPCTP. The supervisors participated and demonstrated key skills in each module with trainees. The programme employed 14 certified standardized patients (SPs) who had graduated from our university and were trained to act as real patients. In this programme, the SPs adopted the role of an angry patient or a patient with malignant cancer who required specialized psychological communication.

The training course included 10 sessions (each session was 3 h in duration). The duration of training was 30 h in total, and the training was completed within 2 weeks. The programme consisted of a 3-h module that focused on theoretical knowledge, a 21-h module focused on communication skills for two-person consultations, a 3-h module focused on communication skills for three-person consultations (when a family relative accompanies the patient) and, last, a 3-h module that assessed the programme effectiveness through a validated evaluation scale administered to the standardized patients (SPs), surgical residents, and supervisors. The evaluation was conducted three times, i.e., before, immediately after and 1 month after the DPCTP, and each evaluation lasted 1 h (see details in Table 1).

**Table 1:** Doctor-patient communication programme for surgical residents

Module	Content	Duration
Module 1 Theoretical course	• Principles of medical ethics and professionalism in doctor-patient communication. • Doctor-patient relationship skills: tone of voice, effective nonverbal communication, eye contact, facial expression, head nods, posture.	3 h
Modules 2–10 Skills learned through practice and feedback		
• Steps: supporting knowledge (0.5 h) → personal experience exchange (0.5 h) → video review (0.5 h) → checklist (0.5 h) → role play (1 h) → feedback (1 h). • The presentation in each module is modified to the trainee’s needs and style based on personal experiences.		
Module 2 Gathering of information during admission and relationship building	• Purpose: to understand both the disease and the patient. • Skills: greet the patient, use a formal address (Nice to see you, Mr......) when communicating within the doctor-patient relationship, use the word “we”, use open-ended questions, do not interrupt the patient. • Checklist: set the stage by asking about the reason for the visit → elicit the patient’s complete story → give information → understand the patient’s perspective → end the encounter → transition to the physical exam.	3 h
Module 3 Interview before surgery and discussion of the treatment plan	• Purpose: to respect the patient’s right to informed consent, explain the disease and agree on a treatment plan with the patient; to explain the cause of a lesion, the possible progress of the disease, and the therapies and surgical procedures that are planned or being conducted. • Skills: discover what the patient knows and thinks, explain effectively, ensure an understanding of the problem. • Checklist: elicit an explanation of the problem from the patient → explain the problem to the patient → ensure the patient’s understanding → agree on a treatment plan with the patient.	3 h
Module 4 Management of angry patients	• Chinese press reports frequently target doctors, patients are usually hostile, and there is a tendency for the patient to blame the doctor. • Skills: show genuine concern and answer all of the patient’s queries. • Checklist: prepare the setting → tell the truth with empathy → gain support from family members and suggest spiritual or cultural support → offer hope to the patient.	3 h
Module 5 Discharge notification	• Purpose: to discuss the importance of good adherence to a regular follow-up schedule before discharge. • Skills: discover what the patient knows and thinks, explain effectively, ensure the patient’s understanding. • Checklist: elicit an explanation of the problem from the patient → explain the problem to the patient → ensure the patient’s understanding → agree on a treatment plan with the patient.	3 h
Module 6 Breaking bad news about a cancer diagnosis and other serious states of illness	• Breaking bad news to patients is a complex and challenging communication task in medical practice; thus, building a good relationship beforehand is especially important. • Skills: create a private environment by closing the door; do not interrupt the patient; provide chairs for everyone; tell the truth with empathy (“I am sorry, but the results are not what we hoped for”); gain support from family members and suggest spiritual or cultural support (“Is there anyone else that you would like to involve?” “Do you have any spiritual, religious, or other beliefs to help you during difficult times?”); offer hope to the patient (“There are many effective treatment options for this disease”); decision making and follow-up. • Checklist: climate prepared → tell the truth with empathy → gain support from family members, as well as spiritually or culturally → offer hope to the patient → discuss decision making and follow-up.	3 h
Module 7 Breaking bad news about perioperative death	• The occurrence of death perioperatively is uncommon; although it is more common during emergency surgery, doctors have to face the facts. The reactions of the patient’s relative are usually hostile, and there is a tendency for the patient to blame the doctor. • Skills: show genuine concern and answer all the queries of relatives. • Checklist: prepare the setting → tell the truth with empathy → gain support from the family members and suggest spiritual or cultural support → offer hope to the patient → discuss decision making.	3 h
Module 8 End-of-life discussion	• To encourage the patient and relieve feelings of pain, anger, and grief while promoting feelings of optimism, surprise, and happiness. • Checklist: prepare the setting → asses the patient’s perceptions and information needs → provide knowledge and respond to emotions with empathy → provide a summary and strategy.	3 h
Module 9 Communication with the patient’s relatives	• Multiple-person interviews are more difficult than interviews with two persons, as they involve more stressful interactions. • To try our best to fulfil the different requirements of family members. • Checklist: set the stage by asking about the reason for the visit → elicit the relative’s queries → give information → understand the relative’s perspective → end the encounter.	3 h
Module 10 Standardized patient evaluation	• To evaluate the effectiveness of the programme from the perspective of SPs. • SPs, residents, and supervisors complete a standardized questionnaire before, immediately after, and 1 month after the programme (3 times). • Each exam lasts 1 h.	3 h

Abbreviations: *DPC*, doctor-patient communication; *SP*, standardized patient

### Outcome

The supervisors completed the extended SEGUE scale [15] to evaluate the competency of surgical residents after their consultations with the SPs. This scale comprises six dimensions, including “setting the stage,” “eliciting information,” “providing information,” “understanding the patient’s perspective,” “closing the encounter,” and “proposing a new treatment.” The extended SEGUE scale consists of 32 questions, with each question rated from 0 to 1, for a total of 32 scores (Additional file 1: Table S1). By completing the visual analogue scale (VAS), the surgical residents and SPs rated their satisfaction levels from 0 to 10 in response to the following question after the encounter: “Regarding the visit you just had with the doctor or patient, please indicate the score.” A score of 0 indicated “poorly satisfied,” and a score of 10 indicated “extremely satisfied.” The extend SEGUE scale and VAS were administered before, immediately after, and 1 month after the 30-h training programme.

### Statistics

Continuous variables with normal distributions were reported as the mean ± standard deviation (SD). Categorical variables were reported as the number (percentage). The Friedman test was used to compare the means of repeated continuous variables. The intraclass correlation coefficient (ICC) was used to determine the consistency between two continuous variables. Significant SEGUE score increases were tested by generalized estimating equations. Statistical analyses were performed using SPSS 22.0 for Windows (SPSS, Inc., Chicago, IL, USA). Values of *P* < 0.05 (two-tailed with Bonferroni adjusted) were considered statistically significant.

## Results

**Fig. 1:**
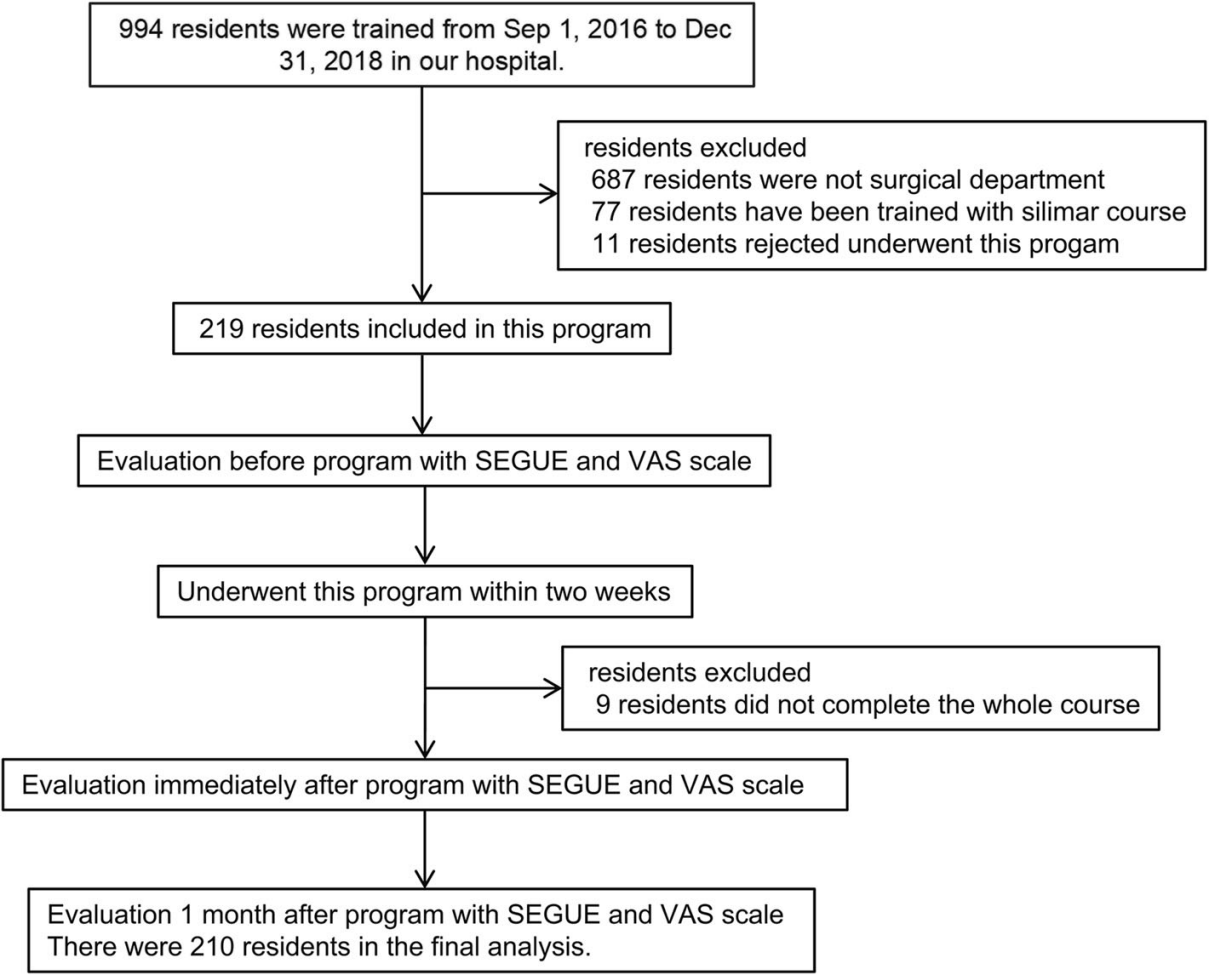
Flow chart of the study. Abbreviations: SP: standardized patient; VAS: visual analogue scale.

A total of 219 surgical residents were originally registered in this programme, 210 of whom completed the entire programme and were included in the final analysis (Fig. 1). Among these, 126 (60.0%) residents were male, and 84 (40.0%) residents were female. The residents were specialized in seven different areas: general surgery (21.0%), paediatric surgery (5.7%), urology (10.0%), neurosurgery (6.2%), cardiothoracic surgery (6.7%), otorhinolaryngology (10.5%), and obstetrics and gynaecology (40.0%). The participants included 66 residents (31.4%) who were in the first year of their residency, 74 residents (35.3%) who were in their second year, and 70 residents (33.3%) who were in their third year (Table 2).

**Table 2:** Baseline data of this cohort

Variable	All residents, n (%) 210 (100.00)
Sex	
Male	126 (60.00)
Female	84 (40.00)
Resident grade	
I	66 (31.43)
II	74 (35.24)
III	70 (33.33)
Specialty	
General surgery	44 (21.00)
Obstetrics and Gynaecology	84 (40.00)
Paediatric surgery	12 (5.70)
Urology	21 (10.00)
Neurosurgery	13 (6.20)
Cardiothoracic surgery	14 (6.70)
Otorhinolaryngology	22 (10.50)

**Fig. 2:**
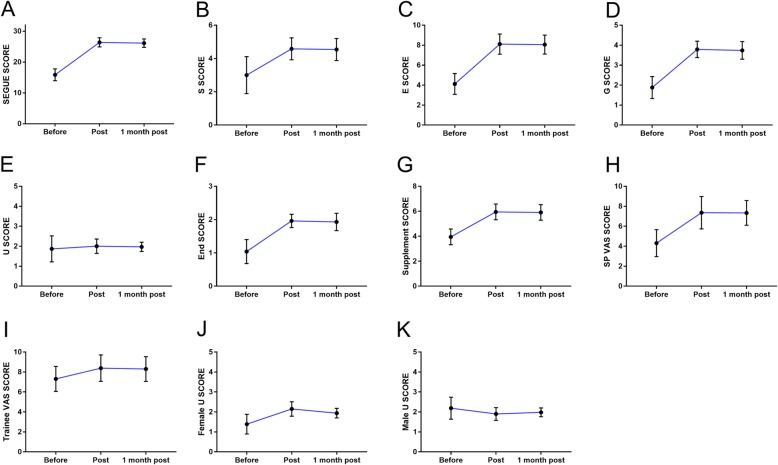
Detailed score changes of the residents, as evaluated by the SEGUE scale and the VAS before and after the programme. Abbreviations: S: Set the stage; E: Elicit information; G: Give information; U: Understand the patient’s perspective; End: End the Encounter; Supplement: Suggest a new treatment plan: SP: standardized patient; VAS: visual analogue scale.

The average scores for the extended SEGUE scale before the programme, immediately after the programme, and 1 month after the programme were 15.88 ± 1.93, 26.40 ± 1.47, and 26.15 ± 1.36 (*p* < 0.001), respectively, indicating significant increase compared to the scores before the programme. Five of the six dimensions of the extended SEGUE scale showed statistically significant score increases (p < 0.001). These dimensions included “setting the stage,” “eliciting information,” “providing information,” “closing the encounter,” and “proposing a new treatment plan.” However, for the understanding the patient’s perspective dimension, no improvement was observed (*p* = 0.542). With respect to this dimension, the results of the subgroup analysis by gender indicated an increase in scores among females and a decrease among males (Table 3, Fig. 2).

**Table 3:** Data from the extended SEGUE scale and the VAS before and after completion of the programme

	Before the programme	Immediately after the programme	1 month after the programme	Statistics	*p* value	Before vs. immediately after the programme	Before vs. 1 month after the programme
SEGUE total score	15.88 ± 1.93	26.40 ± 1.47	26.15 ± 1.36	388.27	< 0.001	< 0.001	< 0.001
Set the stage	3.00 ± 1.11	4.58 ± 0.66	4.54 ± 0.66	371.80	< 0.001	< 0.001	< 0.001
Elicit information	4.12 ± 1.04	8.11 ± 1.01	8.06 ± 0.95	413.17	< 0.001	< 0.001	< 0.001
Give information	1.88 ± 0.55	3.79 ± 0.41	3.74 ± 0.44	411.17	< 0.001	< 0.001	< 0.001
Understand the patient’s perspective	1.87 ± 0.65	2.00 ± 0.36	1.97 ± 0.23	1.168	0.542	0.162	0.450
End the encounter	1.04 ± 0.36	1.96 ± 0.20	1.93 + 0.26	368.54	< 0.001	< 0.001	< 0.001
Suggest a new treatment plan	3.95 ± 0.63	5.95 ± 0.63	5.91 ± 0.62	414.94	< 0.001	< 0.001	< 0.001
SP VAS score	4.31 ± 1.35	7.36 ± 1.62	7.34 ± 1.24	414.78	< 0.001	< 0.001	< 0.001
Trainee VAS score	7.31 ± 1.25	8.39 ± 1.32	8.30 ± 1.24	391.96	< 0.001	< 0.001	< 0.001
Consistency of the VAS between SP and trainee (ICC)	0.26 p < 0.001	0.70 p < 0.001	0.70 p < 0.001				
Subgroup analysis in the understand the patient’s perspective module by gender							
Female	1.39 ± 0.49	2.15 ± 0.36	1.94 ± 0.24	92.85	< 0.001	< 0.001	< 0.001
Male	2.19 ± 0.55	1.90 ± 0.32	1.98 ± 0.22	33.05	< 0.001	0.004	0.054
Both female and male	1.87 ± 0.65	2.00 ± 0.36	1.97 ± 0.23	1.168	0.542	0.162	0.450

Abbreviations: *SP*: standardized patient; *VAS*: visual analogue scale; *ICC*: intraclass correlation efficient

The average VAS scores for the surgical residents before the programme, immediately and 1 month after the programme, were 7.31 ± 1.25, 8.39 ± 1.32, and 8.30 ± 1.24 (*p* < 0.001), respectively. The average VAS scores for the standardized patients before programme, immediately, and 1 month after the programme were 4.31 ± 1.35, 7.36 ± 1.62, and 7.34 ± 1.24 (p < 0.001), respectively. The observed consistency between the SPs and surgical residents was 0.26 (p < 0.001), 0.70 (*p* < 0.001) and 0.70 (*p* < 0.001) before the programme, immediately after the programme, and 1 month after the programme (Table 3).

Significant SEGUE score increases were found both immediately after the programme and 1 month after the programme for the trainees with lower baseline scores. The odds ratios of a significant SEGUE score increase for those with lower and higher baseline scores were 7.017 (p < 0.001) and 11.716 (p < 0.001), respectively. These results were tested by generalized estimating equations and were adjusted by gender, resident grade and specialty (see details in Table 4).

**Table 4:** Generalized estimating equation (GEE) models of significant SEGUE score changes

Significant SEGUE score change (lower baseline vs higher baseline)	β	OR	95% CI	P value
Before vs. immediately after the programme	1.948	7.017	2.816, 17.490	< 0.001
Before vs. 1 month after the programme	2.461	11.716	5.206, 26.365	< 0.001

A lower baseline SEGUE score was defined as a score less than 16 points, which was less than half of the total score (32 points). A higher baseline SEGUE score was defined as a score between 17 and 32 points. A significant SEGUE score change was defined as a difference of 10 points higher than the baseline score

These results were tested by generalized estimating equations (GEEs) and adjusted by gender, resident grade and specialty

Abbreviations: *OR*: odds ratio; *CI*: confidence interval

## Discussion

Residents view medical education as the path to becoming a qualified doctor who is a professional, academic, health advocator, leader, collaborator, and communicator. DPC training programmes are a very important part of post-graduate education to become a qualified doctor. However, the residency system was established in China in 2014, almost a century later than in Western countries. Thus, the provision of effective and comprehensive DPC training programmes for residents is substantially lacking. Moreover, the educational curriculum for medical students at the university level focuses solely on theory.

DPC training programmes were introduced in China approximately 10 years ago, and some training models and assessment instruments were originally derived from Western principles. However, cultural differences and barriers undeniably exist between China and Western countries, particularly in terms of traditional family decision-making (Chinese principles) versus autonomy (Western principles) and an authoritative manner (Chinese doctors) versus patient-centred communication (Western doctors) [16]. Disclosure can also be guided by cultural differences. For example, the East is more community-oriented, while the West is more individualist [17]. Therefore, we should place greater emphasis on adding specific Chinese cultural elements to Western DPC training programmes to adapt them to reflect such differences. Given the frustrating doctor-patient relationship that has been observed in China, an enhanced effective DPC training programme should be a public health priority. However, while the literature contains a few studies that focused on outlining the content of DPC training programmes, there has been no research focused on the concerns of surgical residents in China, in particular. The purpose of this training programme was to enhance doctors’ competence in DPC and to improve patient satisfaction. This is the first prospective study to assess the effectiveness of a modified DPC programme specifically designed for surgical residents in China.

The results of this study demonstrated that the general level of DPC competency among surgical residents in our hospital was relatively low before training, at only 15.88 ± 1.93 (extended SEGUE score), and the satisfaction level of SPs was 4.31 ± 1.35 (VAS score). Both scores were less than half of the total score. However, immediately after completing the DPC training programme, a significant improvement was observed in the competency of surgical residents in DPC (26.40 ± 1.47), as well as in SPs’ level of satisfaction (7.36 ± 1.62), and such improvements were also found at least 1 month after the training programme. However, the results revealed a negative finding with respect to the “understanding the patient’s perspective” dimension, which may have been due to the influence of doctors’ traditional interviewing habits and the fact that doctors in China tend to conceal their emotional expressions from patients. The results of the subgroup analysis by gender revealed an improvement in the competency of female residents with respect to “understanding the patient’s perspective”, which was not found for male residents. These results are in line with the findings of Rao et al., who found that SPs were more satisfied after a consultation with a female resident than a consultation with a male resident. This finding may be attributed to the higher levels of empathy of females [17]. Using the Jefferson Scale of Physician Empathy, Wen et al. found that the average empathy score of Chinese doctors was 109.54. This study involved 1200 doctors, and the scores were less than those of American (120) and Italian (115.1) doctors. Furthermore, female doctors scored higher than males with respect to perspective-taking or, more specifically, compassionate care and the ability to “stand in the patients’ shoes”. Empathy is an aspect of one’s personality and plays an important role in building interpersonal relationships. Therefore, future studies are necessary to further examine this dimension, especially in relation to male surgical residents.

In this study, significant SEGUE score increases were found both immediately after programme (OR = 7.017, *p* < 0.001) and 1 month after programme (OR = 11.716, p < 0.001) for trainees with lower baseline scores. This finding was in line with a previous study by Bylund et al. [18]. They also found that participants with lower baseline scores had larger improvements with both standardized and real patients.

The satisfaction level of both SPs and surgical residents increased significantly after training. The main reason for the SPs’ satisfaction level increase was the improvement in the DPC competency of the surgical residents. In addition, before the training programme, consistency in the satisfaction levels between residents and SPs after medical encounters was very low (ICC = 0.26, *P* < 0.001). However, the consistency increased significantly following the completion of the programme (ICC = 0.70, P < 0.001). The difference in the VAS scores between SPs and surgical residents was smaller after the programme than has been reported previous results. This improvement still existed 1 month after the programme. These results suggested that the SPs’ ratings were more valid than the residents’ ratings, which would indicate a low self-awareness of one’s own communication competence among the residents. After the programme, the surgical residents’ evaluations of their communication competence were more in accordance with those of SPs than before the programme. Similarly, Ha et al. found that many patients reported dissatisfaction with DPC, while many doctors tended to overestimate their communication competency [19]. Tongue et al. revealed that 75% of surgeons believed that they demonstrated satisfactory DPC with patients, while only 21% of patients reported satisfaction with the DPC [20], even though most patients wished to have good DPC. This finding may be attributed to the way in which doctors and patients may have distinct social and cultural characteristics, such as race, gender, culture, socioeconomic status, beliefs, education level, language, expectations, and communication styles. All of these factors may create barriers between doctors and patients.

The doctor-patient relationship has changed. Traditionally, this relationship has been doctor-centred. In this respect, doctors usually spend a substantial amount of time sharing their thoughts with patients, while less time is allocated to listening to patients, eliciting patient concerns or considering the extent to which the patient understands information. Doctors prefer to have an uninterrupted narrative. It is only at the close of a conversation that patients are allowed to ask questions [21]. However, a patient-centred approach, which encourages doctors to focus on respecting patients’ preferences, desires, and values and helping patients regain a sense of control and mastery over their disease or treatment, is accompanied by a high satisfaction level [22].

Good DPC skills include engaging in nonverbal behaviour, listening attentively, showing trust and respect, using open-ended questions, offering sympathy, and empathizing with patients. Although doctors possess different innate talents in the area of communication skills, they can learn and practice these skills, and they are capable of modifying their communication styles [21]. Moreover, doctors should practice such skills to prevent a lapse. As an old Chinese expression says, “I hear and I forget. I see and I remember. I do and I understand” (Confucius, 551–479 BC). The most effective training methods to improve DPC skills were shown to be role play and interactions with standardized patients [1]. Van den Eertwegh et al. also explored a similar DPC training programme for general practitioner trainees [22], which included a five-phase learning model that stressed the value of repeated practice and the importance of integrating this into the trainees’ personal repertoire. The programme in this study was resident-centred, skills-focused, and practice-oriented and adopted a multi-model teaching strategy in small groups, which satisfied these requirements.

This study used a small group strategy in the programme so that the number of participants in each group allowed each learner to benefit from frequent opportunities to practice. This strategy may also facilitate a safe and personal environment to disclose attitudes and feelings. Role play was another strategy used in the programme. The positive effect of role play has been confirmed in a few studies. Trainees may adopt the role of the patient, which fosters insight into how patients are affected during DPC, thereby enhancing an understanding of the patient’s perspective. This also permits repetitive practice of specific communication skills with the ability to make errors in a safe setting. Furthermore, this strategy allows trainees to identify their shortcomings. Practical exercises stimulate the intrinsic motivation of participants, encouraging them to learn how to manage such situations more effectively [23].

Excellent DPC involves five dimensions: effective relationship building, information gathering, understanding of the patient’s perspective, information giving, and good decision-making [15]. The effectiveness of the current programme was evaluated using the extended SEGUE scale, which includes six dimensions and covers the above five dimensions well. The extended SEGUE scale was first administered by Makoul et al. in 2001 [15], and it is in line with the sequence of practical consultation. It not only has high reliability and validity but also is easy to understand. It has been widely used in education and evaluations. Our DPC programme included three modules, namely, “Gathering of information during admission and relationship building”, “Interview before surgery and discussion of the treatment plan” and “Discharge notification”, which were consistent with the routine sequence of clinical practice and covered all dimensions of the extended SEGUE scale.

Most scales used to evaluate DPC competency depend on external observers to judge whether participants were effective or ineffective at communication, as such a method is objective. However, patient satisfaction is also very important, and it is the ultimate goal of DPC. The level of patient satisfaction is determined by the extent to which patient health care needs, expectations, or preferences are met, which contributes to ensuring patients’ better adherence, thus enhancing subsequent treatment outcomes [24]. Thus, this study used a VAS, which was completed by both residents and SPs. In addition, there are clear circumstances in which communication skills that were once learned are easily forgotten. Therefore, assessments were scheduled 1 month after the programme to allow sufficient time to transfer learned communication skills into clinical practice. Repeated practice is necessary and could be improved during the medical process. Delvaux et al. also reported that most of the skills acquired continue to be practised at an excellent level 3 months following training [25]. We conducted longitudinal post-training assessments both immediately after and 1 month after the programme; therefore, the results were more reliable than those of studies that have relied only on immediate post-training assessments.

The first study to evaluate how bad news is delivered to oncology patients in China used the SPIKES model [26], which includes six steps, namely, “setting”, “patient’s perception”, “information need”, “knowledge provision”, “empathetic response to emotions”, and “summary and strategy.” Breaking bad news, such as disclosing a cancer diagnosis to a patient or family, is a dilemma. In Western culture, the approach to breaking bad news is rooted in the patient’s autonomy. However, in Asian cultures, the principle of family decision-making is of utmost importance, with the main reason being the patient’s fear of death, a denial of the cancer diagnosis, and the taboo subject of death. This issue should be appropriately recognized and acknowledged [27]. Therefore, our programme included a module for “Breaking bad news about a cancer diagnosis and other serious states of illness”, which addressed this issue specifically. This module was adapted to Chinese culture and encouraged doctors to deliver bad news with confidence.

Death on the operating table or during the perioperative period is uncommon and usually occurs during emergency surgery, after which doctors are confronted with challenging situations. The reaction of the patient’s relatives is usually hostile, and there is a tendency to place the blame on doctors. In such circumstances, doctors should express genuine concern and answer all queries that relatives may have. Most doctors who have experienced this situation report that it is stressful and that they fear damage to their reputations [28]. As such, our programme included a module for “Breaking bad news about perioperative death”, which addressed this issue specifically and effectively improved this situation. This module was also a modification of the extended SEGUE framework.

Over the past 30 years, numerous strategies and frameworks for end-of-life (EOL) discussions have been developed and implemented with success. Such frameworks include the SPIKES 6-step protocol, the ABCDE Plan, and VitalTalk [29]. A collaborative approach that engages a team of professionals, including doctors, nurses, social workers, and psychologists, can facilitate EOL discussion. Effective teams communicate information and provide support to patients and their families. The team must be united and work together to achieve the same goals. Before a conversation, it is important to have a full understanding of the patient’s illness, and protecting their privacy is also a key point. The patient’s family usually feels loss during this process. As such, we should do our utmost to fulfil the different requirements of multidisciplinary teams. Multiple-person interviews are more difficult than those involving two people, as they involve more stressful interactions [30]. This programme included two modules, namely, “End-of-life discussion” and “Communication with the patient’s relatives”, which addressed this issue specifically and could improve this clinical situation effectively. These modules were also modifications of the extended SEGUE framework.

There are several limitations. First, SPs are widely employed in research, which ensures high experimental control over the conditions; however, in clinical practice, patients vary greatly in terms of their characteristics. Some research has also demonstrated that the use of SPs in training programmes is not a beneficial approach. A study conducted by Lee and Garvin [31] showed that social factors, such as the patient’s education level and financial situation, should be taken into account, as such factors could influence the satisfaction level associated with DPC. Thus, to evaluate the effectiveness of this programme in the future, real patients and real clinical encounters should be studied. In addition, we could further investigate patient adherence, quality of life, and health outcomes. Second, we did not evaluate the long-term effects of this programme, such as the effects 1 year after training; we only evaluated the short-term effects of this programme. Third, the backgrounds of the trainees differed, which may have influenced the outcomes of the training programme. We only carried out a subgroup analysis by gender and did not analyse other types of background variables, such as specialty or resident grade. Fourth, all the participants volunteered to participate in this programme, which might have led to selection bias. However, in this study, almost all surgical residents (95.51%, 229/230) participated in the programme, and only 11 residents left. Fifth, it is necessary to apply other scales to evaluate the effectiveness of DPC. Sixth, future studies should conduct a multi-canter trial to confirm the effectiveness of this programme in China.

## Conclusion

This training programme improved the DPC competency of surgical residents, effectively increased the level of satisfaction of both SPs and surgical residents, and improved the consistency of evaluation between SPs and surgical residents during doctor-patient encounters.

## Additional file

Additional file 1: **Table S1.** Extended SEGUE scale (DOCX 15 kb)

## Data Availability

Not applicable.

## Abbreviations

DPC: doctor-patient communication
VAS: visual analogue scale
