# Supplementary material for: Effectiveness of a modified doctor–patient communication training Programme designed for surgical residents in China: a prospective, large-volume study at a single Centre
Source: BMC Med Educ. 2019 Sep 5;19:338. doi: 10.1186/s12909-019-1776-7 (PMC6727532; doi:10.1186/s12909-019-1776-7)
Supplement: Supplementary file 1 — Table S1. Extended SEGUE scale (DOCX 15 kb) [file 12909_2019_1776_MOESM1_ESM.docx]

| **Questions** | **Yes** 1 | **No** 0 |
| --- | --- | --- |
| **Set the stage** |  |  |
| 1. Greet the patient appropriately |  |  |
| 2. Establish the reason for the visit |  |  |
| 3. Outline an agenda for the visit (e.g., anything else? issues, sequence) |  |  |
| 4. Make a personal connection during the visit (e.g., go beyond medical issues) |  |  |
| 5. Maintain patient privacy (e.g., knock, close the door) |  |  |
| Score |  |  |
| **Elicit information** |  |  |
| 6. Elicit the patient's view of the health problem and/or progress (ideas, concerns) |  |  |
| 7. Explore physical and physiological factors (signs, symptoms) |  |  |
| 8. Explore psychological and emotional factors (e.g., living situation, family relationships, stress) |  |  |
| 9. Discuss alternative treatment options (e.g., self-care, last visit, alternative care) |  |  |
| 10. Discuss how health problems affects the patient's quality of life |  |  |
| 11. Discuss lifestyle issues/preventative strategies (e.g., health risk) |  |  |
| 12. Avoid directive/leading questions |  |  |
| 13. Give the patient an opportunity and time to speak (e.g., do not interrupt) |  |  |
| 14. Listen and give the patient undivided attention (e.g., face the patient, verbal acknowledgement, feedback) |  |  |
| 15. Check/clarify information (e.g., recap, ask “how much”) |  |  |
| Score |  |  |
| **Give information** |  |  |
| 16. Explain the rationale for a diagnostic procedure (e.g., exams, tests) |  |  |
| 17. Discuss the patient’s body and situation (e.g., provide feedback from exams/tests, explain anatomy/diagnosis) |  |  |
| 18. Encourage the patient to ask questions |  |  |
| 19. Adapt to the patient's level of understanding (e.g., avoid/explain jargon) |  |  |
| Score |  |  |
| **Understand the patient's perspective** |  |  |
| 20. Acknowledge the patient's accomplishments/progress/challenges |  |  |
| 21. Acknowledge the waiting time |  |  |
| 22. Express caring, concern, and empathy |  |  |
| 23. Maintain a respectful tone |  |  |
| Score |  |  |
| **End the Encounter** |  |  |
| 24. Ask if there is anything else that the patient would like to discuss |  |  |
| 25. Review the next steps of the treatment plan with the patient |  |  |
| Score |  |  |
| Total score |  |  |
| **For a new or modified treatment/prevention plan** |  |  |
| 26. Discuss the patient's interests/expectations/goals regarding treatment/prevention |  |  |
| 27. Involve the patient when deciding on a treatment plan (e.g., options, rationale) |  |  |
| 28. Explain the likely benefits of the options discussed |  |  |
| 29. Explain the likely side effects/risks of the options discussed |  |  |
| 30. Provide complete instructions for the treatment plan |  |  |
| 31. Discuss the patient's ability to follow the treatment plan (e.g., attitude, time, resources) |  |  |
| 32. Discuss the importance of the patient's role in treatment/prevention |  |  |
| Score |  |  |

**Supplemental Table 1.** Extended SEGUE scale
